# Beyond Set Points: Rhythm-Based Detection of Change in Honey Bee Colony State from Hive Sensor Data

**DOI:** 10.3390/biology15212044

**Published:** 2026-11-09

**Authors:** David Cumin, Rose A. McGruddy, James F. Cheeseman, Michelle A. Taylor, Craig D. Millar, Francesco Merola, Cather M. Simpson, Matthew D. M. Pawley, Logan A. Alexander, Ben H. Paffhausen, Randolf Menzel, Guy R. Warman

**Affiliations:** 1 Department of Anaesthesiology, School of Medicine, University of Auckland, Auckland 1142, New Zealandg.warman@auckland.ac.nz (G.R.W.); 2 New Zealand Institute for Bioeconomy Science Limited, Hamilton 3240, New Zealand; 3 School of Biological Sciences, University of Auckland, Auckland 1142, New Zealand; 4 Department of Physics, University of Auckland, Auckland 1142, New Zealand; 5 School of Chemical Sciences, University of Auckland, Auckland 1142, New Zealand; 6 School of Engineering, Computer and Mathematical Sciences, Auckland University of Technology, Auckland 1142, New Zealand; matthew.pawley@aut.ac.nz; 7 Independent Researcher, Kaikohe 0474, New Zealand; 8 Institute of Biology-Neurobiology, Free University of Berlin, 14195 Berlin, Germany

**Keywords:** actigraphy, automated monitoring, behavioural ecology, chronobiology, circadian rhythms, early-warning indicators, honey bee health, precision apiculture, sensor technology, time series analysis

## Abstract

Disruption of daily patterns of activity, physiology, or social interaction is associated with stress and reduced fitness across species. In social insects such as honey bees (*Apis mellifera*), circadian organisation extends to the colony level, where rhythms emerge from interactions among individual clocks, hive conditions, and environmental cues. Precision apiculture offers a mature sensor toolkit for measuring colony-level variables, and several data streams carry early-warning information about colony condition. Almost all alarms, however, are triggered against set points rather than against the daily rhythm of the signal. We review each sensor technology to establish whether its data have been, or could be, analysed with a chronobiological lens, describing the measurement principle, the achievable temporal resolution and record continuity, the rhythmic signature expected on biological grounds, and any evidence that such a signature has been reported. We then use a synthetic dataset to illustrate the workflow: in this simulated example, rhythm metrics flagged a gradually developing decline approximately three weeks before a conventional threshold alert on the same data. This lead time is a property of the simulation and awaits validation on real colonies. Wherever continuous behavioural monitoring is deployed, rhythm-based metrics may offer a route to earlier, more interpretable detection of change than set point comparisons.

## Simple Summary

Beekeepers and researchers increasingly fit hives with sensors that record temperature, weight, sound, and bee movement at the entrance. Almost all of these systems raise an alarm only when a measurement crosses a fixed limit, such as brood temperature falling below 33 °C. Honey bee colonies work hard to hold such conditions steady, so by the time a limit is crossed the colony is often in serious difficulty. However, it may be more informative to analyse the daily rhythm of the measurements. A healthy colony repeats a strong and regular daily pattern, and a colony under pressure can hold its average conditions near normal for a while, but its daily pattern becomes weaker, less regular, and/or shifted in timing. In this review, we examine each sensor technology used on hives, ask whether its data have the timing and length of record that this analysis needs, and summarise which daily patterns have been reported. Using a synthetic dataset, not a real colony, we then illustrate how rhythm-based measures could flag a slowly developing problem weeks before a conventional temperature alarm. The data and workflow are openly available so the approach can be applied to real hive records.

## 1. Introduction

Concerns over honey bee (*Apis mellifera*) colony losses, which are a direct threat to food security and the stability of ecosystems, have largely driven the development of sensor-based tools for monitoring colony health. This practice, often termed “precision apiculture” or “precision beekeeping”, relies on continuous data collection to track hive conditions and inform colony management.

In nearly every attempt to predict declining colony health, thresholds are set at a single level (a temperature, a mass, a count, or a concentration) or an acceptable range. This is a reasonable default, and it works well for discrete high-impact events such as swarming, queen loss, or sudden population collapse [1,2]. It is less suited to the gradual behavioural shifts that may precede those events. Honey bee colonies are resilient superorganisms that expend considerable effort to hold internal conditions constant. A colony under sustained pressure may continue to defend brood nest temperature within the normal 33 to 36 °C range while reallocating labour away from foraging, grooming, and comb maintenance to do so. Under a set point rule, an alarm will sound only once the defence itself fails, at which point substantial damage may have already occurred and intervention options are limited.

One dimension of behaviour that is routinely recorded in almost all organisms, but seldom analysed, is its temporal structure. Daily rhythms synchronise biological activity and physiology with predictable environmental cycles. Across taxa, disruption of these rhythms is associated with illness or stress [3,4,5]. Neonicotinoids, for example, have been shown to disrupt the circadian rhythms of individual honey bees kept in laboratory conditions [6] and affect the colony in the field [7,8].

A circadian rhythm is generated by an endogenous clock: it persists with a period close to 24 h under constant conditions and is entrained, rather than driven, by environmental cycles. A diel or daily pattern is simply a 24 h periodicity observed in the field, and in a hive record it is the joint product of the colony’s circadian organisation and of direct environmental driving (masking) by light, temperature, weather, and foraging opportunity. Fixed hive sensors record diel patterns, not circadian rhythms as such; separating the two would require constant conditions or explicit modelling of the environmental drivers. Throughout this review we, therefore, use “circadian” for the colony’s internal temporal organisation and its known mechanisms, “daily” or “diel” for what a sensor records, and “rhythm metrics” for quantities computed from the recorded daily pattern. The diagnostic proposal rests on departures of a colony’s daily pattern from its own baseline under comparable environmental conditions, which is why concurrent environmental covariates are indispensable (Section 5.3), and why a change in a daily pattern should be read as a change in colony condition only once environmental driving has been accounted for.

Rhythm metrics are attractive as diagnostic quantities because a colony can serve as its own control: ratios such as relative amplitude, timings such as acrophase, and stability indices are referenced to the colony’s own recent record rather than to a population norm. We hypothesise that such normalised features are less dependent than absolute levels on between-colony differences in size, genotype, hive construction, or sensor placement. This is plausible but, to our knowledge, untested: between-colony variation in amplitude, acrophase, and stability may itself be substantial, and sensor placement affects amplitude more than phase (Section 3.1). We, therefore, state it as a testable hypothesis, and the within-colony baselines of Recommendation 6 are the design that tests it rather than assumes it.

The practical obstacle to rhythm-based monitoring is not, for most modalities, a hardware limitation. Connected hive scales record at intervals of 5–60 min for months at a time; entrance counters, RFID readers, and in-hive temperature loggers routinely operate continuously across seasons; camera systems record for extended periods where power is available. The temporal resolution needed for rhythm analysis is, therefore, already being achieved. What is usually missing is analytical: raw high-resolution time series are commonly reduced to daily summaries or to threshold-crossing events before they are stored or reported, streams from different sensors are not clock-synchronised, and rhythm-derived quantities are seldom computed.

Numerous reviews of precision apiculture and associated sensor technologies already exist, covering the hardware, its specifications, and its performance in the field, and we do not attempt to duplicate them [9,10,11,12,13,14,15,16,17,18]; readers seeking technical detail on a particular sensor are directed there. Our intention for reviewing the sensor technologies is to understand whether a chronobiological lens could be applied to the data, and if there has been any attempt to analyse daily rhythms.

Specifically, in this review we aim to: (i) survey the sensor technologies used in honey bee research and assess, for each, the temporal resolution and record the achievable continuity, and whether rhythm-derived quantities have been reported; (ii) explain why set point interpretation systematically detects change late in systems that actively defend their set points; and (iii) demonstrate a framework using a simulated multi-sensor dataset to quantify potential improvements in detection lead time. We are not proposing that rhythm analysis should replace level-based monitoring but rather that it be computed alongside existing approaches using data that are already being collected.

The reason we develop the argument in a social insect, rather than any other organism, is mechanistic. In a superorganism, the colony-level rhythm is not simply the sum of individual clocks; it is produced by social synchronisation, including substrate-borne vibrational cues and trophallactic contact, and by the collective regulation of the nest environment [19,20,21,22,23]. Thus, colony-level rhythms are measurable with fixed sensors and without tagging individuals; and the synchronisation machinery is itself a candidate point of failure, so a colony can lose temporal coherence while its aggregate output remains within range. The same social organisation also makes the colony rhythm plastic: individual workers move between arrhythmic in-nest tasks and strongly rhythmic foraging according to colony need Bloch & Robinson 2001 [19], so the colony-level rhythm is expected to vary with workforce composition, brood amount, and season even in a healthy colony.

## 2. Methods for the Literature Review

This review is narrative rather than systematic, as there have already been a number of systematic reviews of precision beekeeping technologies. In addition to the papers covered in those reviews, we searched Google Scholar for (“honey bee” OR “honeybee” OR “*Apis mellifera*” OR “colony”) AND (“circadian” OR “diel” OR “daily rhythm” OR “actogram” OR “entrainment” OR “periodogram”) and considered the first 100 results (10 pages) to ascertain whether there has been an application of chronobiological analysis to the data in the context of honey bee monitoring. Additionally, the list of references was fed into a large language model with internet access (Fable 5, Anthropic™) with the prompt “We are writing an academic paper on the use of sensor technology for monitoring honey bee hives and the application of chronobiological analysis to any generated data. Please review the list of references attached and ensure it is complete. Please list any new reference in APA style with a url.”

### Synthesis Approach

Because the included studies differ in design, outcome, and reporting to an extent that precludes meta-analysis, we synthesised each modality narratively against the same seven criteria: (i) temporal resolution, (ii) record continuity, (iii) level of organisation resolved, (iv) behavioural specificity, (v) robustness to environmental noise, (vi) scalability and field readiness, and (vii) demonstrated suitability for rhythm analysis. For each modality, we also record whether validation against an independent behavioural reference was reported.

## 3. Results of the Literature Review—Sensor Technologies

Below, each modality is assessed against the criteria set out in Section Synthesis Approach. Commercial availability is summarised in Table 1; the comparative assessment, including the rhythm audit, is summarised in Table 2.

**Table 1:** Sensor technologies for honey bee colony monitoring: measurement location and commercial availability.

Sensor Type	Monitor Location	Commercial Availability	Example Commercial Tools
Physicochemical	In-hive	Temperature and humidity are widely available; CO_2_ is emerging	Apisense, BeeHUB Sense, BroodMinder, Arnia Perfetta
Gas sensor arrays (e-nose)	In-hive air	None; research stage	—
Hive scales	Under hive	Widely available	BeeMoS, BeeHUB Queen/GPS, BroodMinder-W, Arnia Perfetta
Infrared & thermal imaging	Hive entrance; external; in-hive	IR counters available; full thermal imaging research only	Beezum eHive Beecounter, BeeHero, FLIR ONE Pro
Acoustic & vibrational	In-hive	Acoustic available; vibrational largely research	Hive Heart, BeeHUB Queen/GPS
Video & imaging	Entrance; in-hive	Entrance systems available; continuous in-hive research only	BeeWise (BeeHome), BeeHero
RFID tagging	Hive entrance	Limited; primarily research use	microsensys iID tags
Harmonic radar	Landscape	None; research stage	—
Doppler radar	Entrance; in-hive	None; research stage	—
Lidar	Landscape	None; research stage	—
Capacitive sensors	Hive entrance	None mainstream; prototype/research	—
Electrostatic field (ESF)	In-hive	None; research stage	—

### 3.1. Physicochemical Sensors

Measurement*.* Temperature, humidity, and carbon dioxide (CO_2_) probes report the internal conditions that the colony actively regulates. They are low-cost, low-power, minimally invasive, and typically sample every 1–15 min for months. Healthy colonies hold brood nest temperature between 33 and 36 °C through clustering, flight muscle shivering, fanning, and evaporative cooling [24,25,26,27,28], and relative humidity between roughly 50 and 90% [12]. Compact non-dispersive infrared sensors have made CO_2_ a practical monitoring parameter reflecting colony size, ventilation dynamics, metabolic state, and aspects of forager communication [19,22,29,30].

Rhythmic content demonstrated. All three of the above variables show clear daily cycles [29,31,32,33,34]. Despite this, the daily cycle is often averaged out rather than utilised as a quantity of interest, for example [26,35]. In field records these are diel patterns in the sense defined in Section 1, and the rhythm metrics of Section 4 are computed on them as such.

Predicted signature*.* Because these variables are tightly regulated by the colony, their mean values and daily amplitudes may remain relatively constant despite changes in colony condition. Consequently, their diagnostic value may lie more in the stability of the daily rhythm and its phase relationship to entrance activity and/or external environmental conditions. Specifically: (i) a colony under increasing thermoregulatory load should show rising intra-daily variability in temperature before any change in daily mean; (ii) CO_2_ amplitude should reflect colony metabolic output and is, therefore, expected to decrease ahead of population decline; and (iii) the phase lag between the CO_2_ peak and the entrance activity peak should be stable in a functioning colony and should drift or decouple when ventilation behaviour is no longer matched to foraging load.

Constraints*.* These signals are integrative: they reflect the combined outcomes of many interacting processes and cannot on their own identify the behaviour producing a change [9]. Interpretation is context-dependent, and strong internal gradients make readings sensitive to sensor placement, which matters more for amplitude than for phase. Persistent declines in CO_2_ have been associated with colony collapse, and elevated CO_2_ with pesticide exposure and possible effects of *Varroa* dynamics [36,37], but these are level-based associations.

### 3.2. Gas Sensor Arrays (Electronic Nose)

Measurement. Arrays of partially selective gas sensors, often termed “electronic noses”, sample hive air and respond to the mixture of volatile organic compounds produced by adult bees, brood, comb, food resources, and pathogens. Where a CO_2_ sensor (Section 3.1) reports a single compound, an array output provides a “chemical fingerprint”. Field deployments have discriminated *Varroa*-infested from *Varroa*-free colonies [38,39].

Rhythmic content demonstrated. No rhythm-derived quantities have been reported. However, the signal carries daily structure, as the accuracy with which *Varroa* infestation can be classified from hive air varies systematically with the time of day at which the air is sampled [39]. However, the authors chose to analyse and report periods where the true positive rate for all three infestation categories exceeded the 24 h average rather than analysing the rhythm.

Predicted signature. Hive air composition integrates ventilation, foraging returns, nectar ripening and brood metabolism, each of which follows the colony’s daily rhythm. A healthy colony should, therefore, express a stable daily rhythm. Loss of amplitude or drift in phase of that waveform would be predicted under the same logic as for CO_2_: a colony reallocating labour away from ventilation and foraging changes the timing of air exchange before mean composition moves outside a fixed range. Because the measurement is chemical rather than behavioural, this modality could also separate microbially driven change from behavioural change when paired with an entrance counter.

Constraints*.* Practical constraints are sensor drift, cross-sensitivity to temperature and humidity, and the need for a controlled sampling protocol [40].

### 3.3. Hive Scales

Measurement. Load cells beneath the hive record total hive mass, typically every 15–60 min across seasons. Mass reflects the combined effects of foraging, nectar intake, honey storage and consumption, wax building, and brood rearing [41]. Advances in load cell technology and data integration have made weight a standard component of electronic hive systems [42,43].

Rhythmic content demonstrated. Hive weight is among the most obviously rhythmic hive signals: mass falls as foragers depart and rises as they return, producing a pronounced daily waveform (a diel pattern driven largely by foraging opportunity, and so heavily masked by weather, rather than a direct read out of the colony’s endogenous clock), superimposed on longer-term changes associated with swarming, nectar flow, overwintering, and colony loss [2,44,45,46]. However, published reports generally reduce the series to threshold events, like a 2–3 kg step change indicating a swarm or a sustained decline indicating starvation [45,47,48].

Predicted signature. The daily mass waveform contains at least three separable rhythm parameters that are not routinely reported: the time of the morning inflection (forager departure onset), the acrophase (the time at which a rhythm peaks) of the net gain phase, and the amplitude of the daily excursion normalised to total mass. A colony losing foraging capacity may show a later and less steep morning inflection and a reduced normalised excursion, and that these may appear before net mass loss becomes detectable against seasonal trend.

Constraints. Weight cannot distinguish among the processes contributing to a change, so it is most informative when considered alongside other variables. Rainfall, equipment additions, and manipulations introduce artefacts that must be flagged rather than smoothed. Because absolute mass differs greatly among colonies, normalised rhythm parameters are more transferable than raw amplitudes.

### 3.4. Infrared and Thermal Imaging

Measurement. Unlike single-point probes, thermal imaging provides high-definition spatial distribution of temperature [25,49]. Applications include estimating colony size from cluster heat signatures [50,51], detecting defensive thoracic heating against hornets [52], identifying abnormal brood temperature patterns potentially linked to *Varroa* infestation [53], and monitoring forager traffic [54].

Rhythmic content demonstrated. Infrared entrance beams produce exactly the kind of continuous, low-dimensional count series that rhythm analysis requires, and daily traffic profiles are visible in such data. Full thermal imaging, by contrast, is generally deployed as an intermittent diagnostic rather than a continuous recording, and we found little evidence of rhythm-level analysis of thermal image series.

Predicted signature. Thermal imaging offers spatially resolved rhythms. The centroid and area of the thermally active region likely oscillate daily in a functioning colony, and that loss of this spatial oscillation precedes the mean temperature decline that current threshold rules detect for predicting colony loss, disruptions to foraging patterns, and *Varroa* infestation.

Constraints. Colony size estimates from heat maps are indicative rather than diagnostic. The non-invasive advantage is not always retained: brood mapping for *Varroa* has required hives to be opened and frames removed [53]. Hardware cost is high, some applications need multiple cameras per hive [50], and entrance infrared requires a narrow field of view that complicates setup [54]. Detectable thermal change ahead of swarming occurs only once departure preparation is underway [49], which limits its warning value.

### 3.5. Acoustic and Vibrational Sensors

Measurement. Microphones and vibration detectors (accelerometers and piezoelectrics) have been combined with machine learning for behavioural classification [1,55,56].

Rhythmic content demonstrated. There is a definite daily rhythm of hive sound [34,57,58]. Substrate-borne vibrations have also been identified as social cues capable of entraining circadian rhythms, implicating them in social synchronisation of the colony clock [23]. This makes vibration both a readout of rhythm and part of its mechanism. Otherwise, acoustic work has concentrated on discrete signals: queen piping and quacking during succession [59,60], pre-swarm worker sounds [61,62], agitation during robbing, and baseline shifts under *Varroa* infestation or pesticide exposure [63,64,65]. Vibrational sensors have been used to assess queen presence and swarming readiness [66] and to detect begging signals and colony-wide vibration signals [67,68].

Predicted signature. Because acoustic signals contain information across multiple frequency bands, rhythm analysis can be performed separately for each band and for patterns over multiple bands, potentially revealing behavioural changes that would be obscured in a single measure of acoustic amplitude. Different bands (or combinations of bands) carry different information, and the phase relationship among bands is probably a more specific indicator of colony state than total acoustic power. Loss of coherence between bands could indicate, for example, that the colony is no longer coordinating ventilation with foraging.

Constraints. Data volume is large; airborne signals are susceptible to wind, rain, and machinery noise [66]; and vibrational data depend strongly on frame material and sensor mounting, so each installation requires its own calibration and there is no single, standardised healthy baseline. Published analysis uses short windows, sometimes only up to one or two days, which does not produce enough data for rhythm-stability assessment.

### 3.6. Video and Imaging Systems

Measurement. Cameras at the hive entrance record forager movement and behaviours such as pollen return, grooming, or swarming [69,70,71]. Restricted pathways or tunnels simplify tracking and support estimates of foraging rate, pollen return, and *Varroa* infestation [72,73,74,75]. Stereo and multi-camera systems reconstruct three-dimensional flight trajectories at the entrance, allowing flight speed, curvature, and hovering to be quantified [76,77]. Internal recordings are less common because of lighting, spatial, and maintenance constraints, but they have been used in laboratory and field settings to capture bee behaviours [78,79,80], to detect *Varroa* [81], and video is widely used to validate other sensors, such as entry/exit detectors and the ESF recordings (see Section 3.11) [82,83,84].

Rhythmic content demonstrated. Entrance video yields continuous count and trajectory series that are, in principle, ideal for rhythm analysis, and daily traffic patterns are evident in published traces. One preprint article has proposed an automated tracking system that reportedly showed a circadian pattern in information transfer between individual bees [85]. However, we found little published analysis of video-derived series as rhythms over multi-week records, which we attribute to storage and processing cost rather than to any property of the signal.

Predicted signature. Video can deliver rhythms of behaviourally specific metrics at colony scale, such as the daily profile of pollen-laden returns relative to total returns. The ratio of pollen-bearing to total returning foragers will have its own acrophase, reflecting brood demand, and changes in daily profile may provide an earlier indication of changing brood status than total forager traffic alone. Three-dimensional trajectory data additionally permit rhythms of flight performance (e.g., speed and curvature) rather than merely flight frequency, and changes in these metrics may indicate declining forager performance.

Constraints. High bee density causes occlusion and frustrates some automated detection techniques. Internal recording requires lighting: visible light may alter behaviour, red light is a partial compromise [86], and infrared alternatives trade spatial resolution and contrast, with sensitivity to wax and propolis accumulation. Outdoors, variable illumination, rain, wind, and obstruction degrade tracking [87]. Power and storage demands have historically limited deployment in off-grid locations, although on-device inference may be a solution.

### 3.7. Radio Frequency Identification (RFID) and Harmonic Radar

Measurement. Radio Frequency Identification (RFID) measurements involve affixing passive tags on the thorax of individual bees. Those tags are read by sensors at the hive entrance, logging exit and re-entry times to record trip frequency, trip duration, lifetime activity budgets, and return rates [88,89,90,91,92]. Harmonic radar is a spatial tracking method where a small antenna stuck to the thorax of individual bees re-radiates an emitted signal, allowing flight paths to be reconstructed to about 1 km [93,94,95]. Its natural outputs are trajectories, navigational decisions, and search behaviour; it has been central to demonstrating time-compensated cognitive maps and the inference of food location from waggle dance information [96,97]. RFID answers when and how often bees forage, while harmonic radar answers where and by what route.

*Rhythmiccontent demonstrated.* RFID has the strongest track record of any modality for analysis of behavioural timing across the lifespan, which is close to rhythm analysis in method even where it is not framed that way. Automated breakpoint procedures applied to lifelong RFID records have been used to detect the age at onset of foraging and to quantify ontogenetic shifts in time activity patterns [98], sleep patterns [99], and lifespan and pre-foraging stage have been characterised from the same kind of data [100]. RFID has also been used to quantify sublethal pesticide effects on foraging and homing [101,102] and pathogen effects on forager performance [103]. Time-of-day structure in these datasets is typically present but usually analysed as a covariate rather than as the outcome.

Predicted signature. In addition to the aforementioned life-cycle analysis, the distribution of individual foraging onset times, rather than their mean, is probably more diagnostic because a colony under stress may show increased between-individual dispersion in onset time and a flattening of the population activity profile, reflecting loss of social synchronisation, before mean trip frequency changes appreciably.

Constraints. Neither method observes in-nest social behaviour, brood care, or collective dynamics directly. RFID tagging is labour-intensive, and there are continuing concerns that the tags or aerials alter behaviour [84,104]. This approach is totally impractical outside the intensive research setting. Harmonic radar is generally limited to a few individuals at a time and requires specialised outdoor equipment. Both are best regarded as research instruments. Importantly, forager return statistics reflect mortality that has already occurred, which makes them lagging rather than leading indicators of colony condition [8,105].

**Table 2:** Comparative assessment of sensor modalities against the seven criteria. The final column records whether rhythm-derived quantities have been reported from the modality. Note that temporal resolution is widely adequate, but rhythm-derived quantities are reported from very few modalities.

Sensor Modality	Level Resolved	Typical Sampling Interval	Continuous Multi-Week Record	Behavioural Specificity	Robustness to Environmental Noise	Scalability & Field Readiness	Suitability for Rhythm Analysis	Rhythm-Derived Quantities Reported to Date
Physicochemical	Colony	1–15 min	Yes, routine	Low—integrative	High	High; low cost, low power	High—best-established rhythmic content; ideal coupling reference	Daily cycles described [29,31,32,33]; rarely quantified as rhythm parameters in publications.
Gas sensor arrays (e-nose)	Colony	Minutes	Possible; drift-limited	Moderate—chemical fingerprint of colony state	Low—moderate (drift; cross-sensitivity)	Low; research prototypes	Moderate—multivariate signal with demonstrated diel structure; drift correction required	Time-of-day dependence of diagnostic accuracy reported [39]
Hive scales	Colony	15–60 min	Yes, routine	Low—mass integrates many processes	Moderate (rain, manipulation artefacts)	High; commercially mature	High—pronounced daily waveform, under-analysed	Daily weight waveform modelled [44]; operationally reduced to thresholds
Infrared entrance counters	Colony	<1 min	Yes	Low—counts only	Moderate (narrow field of view needed)	High	High	Daily traffic profiles shown; rhythm parameters rarely reported
Thermal imaging	Colony (spatial)	Intermittent in practice	Rarely	Moderate—spatial, not behavioural	Moderate	Low; hardware cost, multiple cameras	Moderate—unique spatial-rhythm potential, currently unrealised	None identified
Acoustic sensors	Colony	Continuous audio	Possible; storage-limited	Moderate—signal-specific with classification	Low (wind, rain, machinery)	Moderate	Moderate—high—clearest existing precedent; analytically demanding	Colony daily rhythm recognised from hive sound [57]
Vibrational sensors	Colony	Continuous	Possible	Moderate—specific social signals	Low (hive- and mount-dependent)	Moderate; per-install calibration	Moderate—both readout and mechanism of synchronisation	Substrate vibration shown to entrain rhythms [23]
Video (entrance)	Individual & colony	Frame rate	Storage- and power-limited	High—behaviour-specific	Low (light, rain, occlusion)	Low—moderate; power and storage	Moderate—high—only route to rhythms of specific behaviours at colony scale	Little multi-week rhythm analysis identified
Video (in-hive)	Individual	Frame rate	Rarely	High	Low (lighting alters behaviour)	Low	Low—moderate—lighting confounds rhythm measurement	None identified
RFID	Individual	Event-based	Yes, lifespan-scale	Moderate—timing, not behaviour type	High	Moderate; tagging effort	High for individual timing; lagging indicator of colony state	Lifelong timing structure and ontogenetic shifts analysed [98,100]
Harmonic radar	Individual	Trajectory-based	No	High—spatial navigation	Moderate	Low; cost, one bee at a time	Low—record length inadequate for rhythm analysis	None identified
Doppler radar	Colony	Continuous	Yes	Low—aggregate motion	Low—moderate (vegetation, rain)	Moderate; portable, solar-capable	Moderate—high—simple continuous activity index	None identified
Lidar	Landscape (flight activity)	Continuous transects	Not yet demonstrated	Moderate—flight activity and wingbeat signature	Moderate (weather-dependent)	Low; research instrument	Potentially moderate—landscape-scale daily flight waveform; colony attribution difficult	None identified
Capacitive counters	Colony	<1 min	Yes, months	Low—coarse traffic	High (light- and weather-insensitive)	High; low cost, low power	High—best current match to rhythm-analysis requirements	Multi-day foraging onset/offset shifts described [86]
Electrostatic field (ESF)	Colony (localised)	Continuous	Not yet demonstrated in field	High—specific in-nest behaviours	Moderate; unvalidated in field	Low; research stage, spatial coverage unresolved	Potentially high but unproven; classification and coverage unresolved	None identified

### 3.8. Doppler Radar

*Measurement.* Doppler radar detects motion from frequency shifts in reflected signals, without tagging or visual access, and has been used to estimate entrance activity from bees moving toward or away from the antenna [106]. Systems have been deployed alongside accelerometers to capture swarming, robbing, and defensive responses [107], and internally-focused micro-Doppler approaches can detect vibration patterns associated with general activity and queen presence [108].

*Rhythmic content demonstrated.* Doppler output is a continuous activity index, which is the simplest possible input to rhythm analysis, and sensor units are portable and can be solar-powered. However, we are not aware of any multi-week rhythm analysis of Doppler hive data.

*Predicted signature.* Given that Doppler integrates all motion into one channel, its value is in period and phase rather than in behavioural specificity. Doppler-derived activity likely yields acrophase and interdaily stability estimates closely comparable to those from capacitive or infrared counters, and agreement between the two could establish Doppler as a low-cost, contact-free substitute where entrance modification is undesirable.

*Constraints.* Individual behaviours and social interactions cannot be isolated. Wind-blown vegetation, passing animals, and rainfall introduce noise and reduce accuracy [109]. Furthermore, radar operates in frequency bands whose effects on insect physiology are not fully characterised [110], which warrants caution in long-term deployment near colonies.

### 3.9. Light Detection and Ranging (LiDAR)

*Measurement.* LiDAR non-invasively detects insects in flight. Polarisation LiDAR has been demonstrated on free-flying honey bees, discriminating bees from background over hundreds of metres during landmine detection trials [111]. Entomological LiDAR more generally counts insect transits along a fixed transect and resolves wingbeat frequency modulation, which supports coarse taxonomic separation [112]. It is the only modality in this review that measures flight activity at landscape scale without instrumenting either the hive or the bee.

*Rhythmic content demonstrated.* Entomological LiDAR data from other taxa have shown diel flight activity cycles over multi-day deployments [112], so the temporal resolution and record length required for rhythm analysis are within demonstrated capability.

*Predicted signature.* The aggregate daily flight waveform of the colonies likely shows onset after dawn, weather-gated midday structure, and cessation before dusk. This is the same waveform an entrance counter would show, but it is measured across the foraging landscape and may contain direction of flight also.

*Constraints.* Attribution to a focal colony is the central difficulty, and weather is a strong masking variable.

### 3.10. Capacitive Sensors

*Measurement.* Capacitance sensors at the entrance generate a low-power electric field and register brief changes as bees pass through, giving time-stamped counts without tagging or visual tracking [82,113].

*Rhythmic content demonstrated.* Capacitive sensors can measure colony-level entry activity without the need to label individual bees and with lower power consumption than cameras. Performance is largely insensitive to light and weather, and continuous operation over weeks to months is routine; entrance traffic analysed across multiple days shows shifts in foraging onset and offset and in activity level [86]. Rhythm parameters are nonetheless seldom reported explicitly.

*Predicted signature.* Capacitive count series may show rhythmic activity patterns, and a reduction in amplitude of the daily signal may indicate the onset of *Varroa*-associated or nutritional stress well before total daily counts fall outside their seasonal range.

*Constraints.* Behavioural specificity is low: forager roles, flight purpose, and, depending on configuration, direction of travel cannot reliably be distinguished. Overlapping passage during periods of high traffic can lead to undercounting, and accumulation of bees at the hive entrance during overheating or early swarming produces a sustained baseline shift that corrupts counts [114]. Capacitance, therefore, provides a coarse activity measure, which is adequate for phase and stability estimation but not for behavioural attribution.

### 3.11. Electrostatic Field (ESF) Sensors

*Measurement.* ESF sensors detect fluctuations in local electric fields produced by charged, moving bees. Charge accumulates through friction with air during flight and through contact with hive surfaces and other individuals [83], and wing vibration, abdominal shaking, and walking generate localised field fluctuations. These fields are detected by nearby individuals and influence behaviours including the waggle dance, fanning, and inhibitory stop signals [83,115]. ESF sensing operates in complete darkness without artificial lighting or visual access and can identify specific in-nest behaviours such as waggle dancing, fanning, and potentially tremble, grooming, and shaking movements from their characteristic time-frequency signatures [116].

*Rhythmic content demonstrated.* ESF sensing is at an early stage of development and, to our knowledge, no multi-week ESF record has been published.

*Predicted signature.* If in-nest behaviours can be classified reliably over long records, ESF would provide something no other modality currently offers: rhythms of specific social behaviours measured inside the nest without disturbance. The waggle dance rate may have a pronounced daily acrophase preceding the entrance activity acrophase, and that fanning rate has a distinct thermally driven phase, so that the lag between these two behavioural rhythms would index the colony’s allocation of effort between foraging and thermoregulation. Further, behavioural markers from ESF may well be expressed in signal-specific spatial and temporal distributions, allowing us to test the possibility of localised colony clock(s).

*Constraints.* There is no commercial system, no established performance data across hive materials, colony sizes, or seasons, and no independent validation of behavioural classification accuracy under field conditions, despite a large amount of data already collected [116].

### 3.12. Comparative Synthesis

Adequate temporal resolution is widespread in the sensors currently available in precision apiculture. Physicochemical probes, hive scales, capacitive and infrared entrance counters, RFID, and Doppler radar all sample at intervals well inside the range required to resolve a 24 h cycle, and all are capable of continuous operation over the multi-week records that rhythm stability assessment requires. On the criterion that matters most for circadian work, the deployed base is already adequate.

Furthermore, rhythm-derived quantities are reported from very few of the current datasets. Series are often reduced to daily means, maxima, and minima, or to threshold-crossing events, before analysis. Where rhythm has been analysed directly, such as colony rhythm recognised from hive sound [57], entrainment by substrate-borne vibration [22], temperature and CO_2_ [32], or timing structure in lifelong RFID records [98], it has generally been treated as a biological question in its own right rather than as a diagnostic instrument.

Viewed across Table 1, Table 2 and Table 3, the technical characteristics point in one direction. The modalities that best satisfy the requirements of rhythm analysis (sub-hourly sampling, negligible drift over weeks, low power, insensitivity to lighting and weather, and routine season-long deployment) are the simplest and least expensive: temperature and humidity probes, hive scales, and capacitive or infrared entrance counters. The more sophisticated modalities, which offer greater behavioural specificity, are precisely those whose power, storage, lighting, or calibration demands have so far prevented the long continuous records that rhythm analysis needs. The data best suited to the approach we propose are, therefore, already being collected at scale, which underlines our central conclusion that the barrier is analytical rather than technological.

## 4. A Worked Demonstration on Simulated Data

To illustrate how data might be analysed through a chronobiological lens, we generated a synthetic multi-sensor dataset representing two hives and applied both set point and rhythm-based analyses to it. The example is a demonstration of the workflow and of the logic set out in Section 5.1, and it is circular by construction: the perturbation is specified in the rhythm domain (amplitude, phase, envelope, and day-to-day variability of entrance activity), so the rhythm metrics were bound to respond to it, and the temperature stream is specified to fail late, so the set point rule was bound to respond late. The detection lead time it yields is, therefore, a property of the simulation, not an empirical finding. Its purpose is to show what rhythm-based monitoring might look like in practice, to make the mechanism of Section 5.1 concrete, and to provide an analytic framework that could be applied to real hive records.

### 4.1. Methods

We generated 70 days of data at 5 min resolution (288 observations per day) for two colonies, a control and a perturbed colony, sharing a common weather record. Two streams were simulated for each: entrance activity (counts per 5 min, Poisson-distributed about a unimodal daily envelope with a 13.5 h acrophase) and brood nest temperature (regulated around 34.8 °C with a small daily oscillation). A shared multiplicative day-quality factor represented weather-driven masking of foraging. Both colonies carried day-to-day random effects in amplitude and phase.

From day 30, the perturbed colony was subjected to a gradual perturbation ramping linearly over 25 days, comprising four simultaneous changes: a 55% reduction in activity amplitude, a 2.2 h delay in acrophase, broadening of the daily activity envelope with loss of night-time consolidation, and a progressive increase in day-to-day variability of both amplitude and phase, representing loss of social synchronisation. Brood temperature was allowed to fail only late, from day 52, reflecting the compensation mechanisms previously discussed.

Simulated data were generated with a custom Python (version 3.14.4) script. Analysis was conducted in AnCiR (v β.74.1; https://ancir.pages.dev/; https://doi.org/10.5281/zenodo.19340642; accessed on 21 September 2026). The data generation script, the simulated dataset, and the AnCiR session that reproduces every figure are archived on Zenodo (https://doi.org/10.5281/zenodo.22884569).

Rhythm metrics were computed on rolling 7-day windows advanced in daily steps. For each window, we estimated four rhythm metrics: cosinor relative amplitude and acrophase [117], and interdaily stability and intradaily variability [118]. A baseline was established from windows ending on or before day 28, and control limits set at the baseline mean ± 3 SD. An alert was raised when a metric fell outside its limits for three consecutive windows. For comparison, a conventional threshold alert was raised when brood nest temperature first fell below 33 °C.

### 4.2. Results

**Figure 1:**
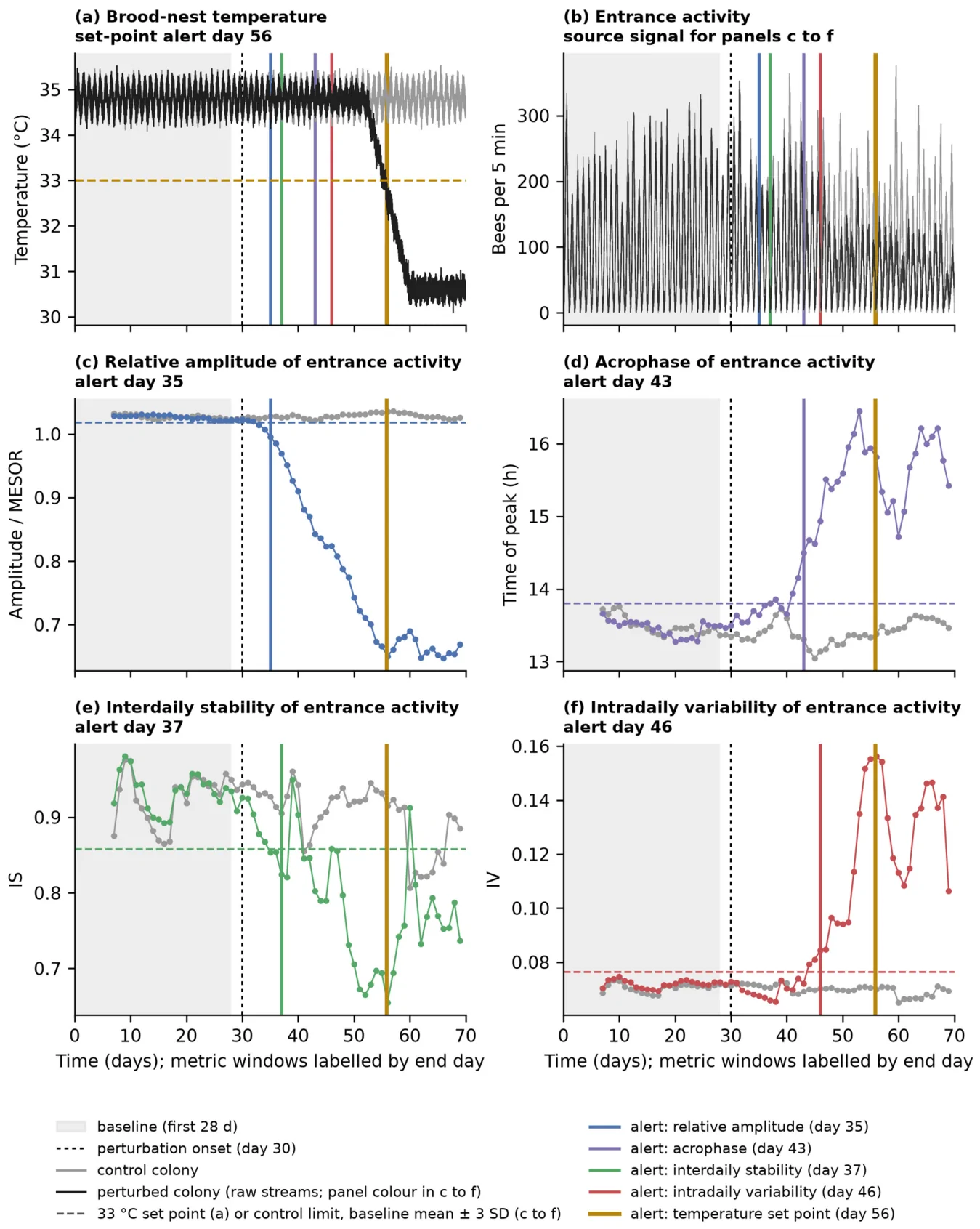
Simulated demonstration of rhythm-based versus set point detection on the control (grey) and perturbed (black or coloured) colonies, all panels on one time axis. (**a**) Brood nest temperature, with the 33 °C set point (dashed horizontal line); the perturbed colony defends temperature near 34.8 °C until day 52, and the set point alert fires on day 56. (**b**) Entrance activity (bees per 5 min); the perturbed colony’s daily amplitude shrinks after perturbation onset while the control colony keeps its rhythm. (**c**–**f**) Rolling rhythm metrics, all computed on entrance activity in 7-day windows advanced daily and labelled by their end day: (**c**) relative amplitude of entrance activity (cosinor amplitude divided by MESOR), alert on day 35; (**d**) acrophase of entrance activity (time of peak activity, hours), alert on day 43; (**e**) interdaily stability of entrance activity, alert on day 37; (**f**) intradaily variability of entrance activity, alert on day 46. In (**c**–**f**), the dashed horizontal line is the control limit (baseline mean ± 3 SD from windows ending on or before day 28), and an alert is raised when the metric lies outside its limit for three consecutive windows. In every panel, the shaded region is the 28-day baseline window, the dotted vertical line marks perturbation onset (day 30), and coloured vertical lines mark the first alert from each rhythm metric and from the temperature set point. The control colony crosses no limit in any panel.

Figure 1 shows the two raw streams and the four rhythm metrics on a common time axis. Brood nest temperature in the perturbed colony (Figure 1a) remained within the normal range for more than three weeks after perturbation onset because the simulated colony, like a real one, was specified to defend temperature until late in its decline; the 33 °C set point was first crossed on day 56. Entrance activity (Figure 1b) tells a different story: the daily amplitude of the perturbed colony began to shrink from day 30, and its night-time activity increased, while the control colony kept its rhythm throughout. Figure 1c–f show how the four rolling rhythm metrics, all computed on entrance activity, responded. Relative amplitude (Figure 1c) gave the earliest sustained alert, on day 35, five days after onset and consistent with a 7-day analysis window. Interdaily stability (Figure 1e) followed on day 37 as the day-to-day reproducibility of the waveform degraded, acrophase (Figure 1d) on day 43 as the accumulated phase delay exceeded its control limit, and intradaily variability (Figure 1f) on day 46 as the within-day profile fragmented. The control colony crossed no limit on any metric (grey series), so the false alarm count over the 70-day record was zero. Table 4 summarises the alert days. In this synthetic example, the earliest rhythm-based alert preceded the conventional threshold alert by 21 days. This interval reflects the perturbation we chose to simulate (a 55% amplitude loss ramping over 25 days, with brood temperature defended until day 52). Any rhythm-based alarm timing needs to be established empirically.

Amplitude responded first because amplitude reduction was the largest component of the simulated perturbation. The order in which the remaining metrics responded reflects how quickly each component of the perturbation accumulated relative to its baseline variability: loss of day-to-day stability as the between-day variance grew, then phase drift as the 2.2 h delay accumulated, and intradaily variability last, as the within-day fragmentation developed most gradually. In the field, this ordering may be informative, as different stressors could be expected to produce different patterns of change across the metrics.

## 5. Discussion

### 5.1. Why Set Point Monitoring Detects Change Late

Current monitoring is technically advanced but conceptually homeostatic. It assumes that a healthy honey bee colony remains within a fixed, stable range, and it programmes alerts to fire when a measurement crosses an absolute limit. The difficulty is that colonies are resilient, and expend considerable energy remaining stable while under pressure [119]. Moreover, the circadian rhythms suggest that organisms maintain homeorhesis [120], rather than homeostasis, and so measuring the changes in rhythms may better reflect the underlying biology. Absolute values may, therefore, be a late indication of trouble.

The brood temperature example confirms this. A system that alerts when hive temperature falls below the 33–36 °C range is likely identifying a terminal state. Temperatures depart from the range when the brood nest has been lost or worker density has fallen below the minimum required for thermoregulation, that is, once the failure has already happened. The same logic applies across modalities: a 3 kg step loss in hive mass identifies a swarm that has departed; a spike in acoustic power identifies agitation already underway; a fall in forager return rate identifies individuals that have already died. Each of these is a lagging indicator by construction because each is defined by the crossing of a boundary that the colony has been actively defending.

This review and analysis suggests that a more sensitive approach may be to monitor the behavioural effort required to maintain stability rather than the stability itself. A colony under pathogen or nutritional pressure may keep the nest warm, but it does so by reallocating labour away from foraging, grooming, and comb maintenance. Such reallocation changes when activities occur and how tightly they are coordinated long before it changes what the aggregate sensor reads. Rhythm parameters (amplitude, phase, day-to-day reproducibility, and the phase relationships among concurrently recorded streams) are possibly a more direct measurement of that coordination and so may act as an “early warning” signal [121,122].

Rhythm metrics are not universally superior, and we do not propose them as a replacement for threshold rules. As the rhythms are computed over multi-day windows, they are slow to respond to acute, discrete events, such as swarming, robbing, wasp attack, hive tipping, or theft, all of which are detected faster and more reliably by threshold or step change rules on the raw series. Rhythm metrics also have their own failure modes, principally environmental masking, which is why concurrent environmental covariates are essential (Recommendation 4), as is biological variability, discussed next.

Biological variability is a second and less tractable source of false alarms. The honey bee circadian system is strongly socially regulated. Young nurse bees tend brood around the clock with weak or absent behavioural rhythms and attenuated clock gene cycling, foragers are robustly diurnal, and workers switch between these states, in both directions, according to the needs of the colony [19,123,124]. Social cues can override photic entrainment altogether [125]. The rhythm that a fixed sensor records is, therefore, a weighted mixture of these individual patterns, and its amplitude, phase, and stability will shift whenever the mixture does: as the forager-to-nurse ratio changes with brood area and colony size, after swarming or requeening, during a nectar flow, and across the season as the colony moves from brood rearing to a broodless winter cluster whose thermoregulation is governed by different mechanisms [126]. None of these changes is a sign of ill health, yet each would register on the metrics used in Section 4. Within-colony baselines and control limits (Recommendation 6) absorb some of this variation because the colony is compared with its own recent past rather than with a population norm, but a 28-day baseline cannot absorb seasonal transitions or management interventions. In practice, rhythm-based alerts will need to be interpreted against a record of colony size, brood area, and beekeeper manipulations, and the expected seasonal trajectory of the metrics will need to be characterised empirically before departures from it can be read as warnings.

The worked example in Section 4 uses synthetic data and is circular by design: the perturbation was specified in the rhythm domain, so the rhythm metrics were bound to respond, and the comparator variable was specified to fail late. The three-week lead time it reports is a consequence of what we specified, not evidence that such a lead time is observed in the field, and the ordering of the four metrics is likewise a property of the simulated stressor. What the example does show is the mechanism by which a colony that defends its set point can change its temporal organisation long before the defended variable moves, and the workflow by which such a change would be detected. Whether real stressors act on colony rhythms in this way, and with what lead, can only be settled with real records analysed against pre-specified rules (Recommendation 7). We have provided the data generation script and the complete analysis session so that the workflow can be applied to real records.

### 5.2. Generality Beyond Honey Bees

Honey bee colonies are a useful model system for rhythm-based monitoring for four specific reasons: (i) colonies occupy a fixed nest site, so fixed sensors can record continuously for months without recapture or battery replacement; (ii) the superorganism structure means colony-level rhythms are measurable in aggregate signals without tracking individuals; (iii) colonies are strongly entrained by light and temperature, giving a well-defined expected rhythm against which departures can be assessed; and (iv) a large instrumented base already exists, so the analytical framework can be applied retrospectively to archived records.

Importantly, the metrics and approach are not bee-specific. Interdaily stability, intradaily variability, and relative amplitude were developed for human rest activity actigraphy [127,128], and rhythm analysis is well-established in many fields [117].

The use of chronobiological analysis requires data to span many days, so short deployments are unsuitable. A disturbance-free baseline must also be obtainable, and masking covariates must be measurable, which is straightforward at a fixed apiary and much harder for a hive that is moved for pollination or a free-ranging animal moving through heterogeneous conditions.

The approach will also transfer poorly to some systems. Species with weak circadian organisation, animals undergoing hibernation, those whose activity is dominated by episodic events, such as long-distance migration, and those whose rhythms reorganise seasonally in ways that are themselves adaptive will all generate rhythm changes that are normal rather than pathological. In such systems, the metrics remain computable, but their interpretation as health indicators does not follow.

### 5.3. Recommendations

Based on this synthesis, we propose seven recommendations for data collection, analysis, and reporting, presented in order of increasing complexity of implementation.

• Report sampling interval and total record duration in every sensor-based study. These determine what temporal analysis is possible and are frequently omitted.
• Archive the raw high-resolution series, not daily summaries or threshold-crossing events. Reduction is irreversible, and it discards precisely the structure that rhythm analysis needs.
• Synchronise clocks across sensors to a common network time source and record timestamps in UTC with an explicit offset.
• Record environmental and colony covariates concurrently (weather at the apiary, colony size, brood area, and all beekeeper manipulations), at the same temporal resolution as the behavioural streams where possible.
• Report at least one rhythm metric (relative amplitude, acrophase, or interdaily stability) alongside conventional level-based summaries. This is a small addition to any analysis and would rapidly build the comparative base that currently does not exist.
• Establish within-colony baselines and set control limits from them rather than from population set points.
• Benchmark lead time explicitly against a threshold rule computed on the same data, using pre-specified decision rules and published code.

## 6. Conclusions

Circadian rhythms offer a powerful but underused framework for interpreting behavioural data in ecological monitoring, and honey bee colonies, whose activity reflects internal physiological processes and strongly cyclic external conditions, are a well-suited system in which to demonstrate it. Four insights emerge from this review. First, the sensing problem is largely solved: fourteen of the fifteen modalities surveyed already achieve the temporal resolution needed for rhythm analysis, and the simplest and cheapest of them routinely produce the season-long continuous records that it requires. Second, the analytical opportunity is largely unexploited: rhythm-derived quantities have been reported from only a handful of modalities, and raw series are routinely reduced to daily summaries or threshold events before the rhythmic structure can be examined. Third, set point alarms are late by construction in a system that actively defends its set points, so monitoring the behavioural effort required to maintain stability, rather than stability itself, is the more sensitive strategy; our synthetic example illustrates that mechanism and the workflow for detecting it, not the lead time to expect in real colonies. Fourth, the principal obstacles to adoption are not technical but biological and procedural: the plasticity of the colony rhythm with workforce composition, brood area, and season means that normative trajectories must be characterised empirically, and lead times must be benchmarked against threshold rules on the same real data using pre-specified decision rules. The seven recommendations in Section 5.3 are intended to make that validation possible with data that are already being collected. Although this review focuses on honey bees, circadian patterns structure behaviour in many species, and the same reasoning applies wherever animals are monitored continuously, linking field observation to physiological state in ecological research, health assessment, and animal management. The proposition is simple and testable: watch the rhythms in different signals, and the harmony among them, not only the levels.

## Figures and Tables

**Table 3:** Measurement resolution and drift characteristics of each sensor modality that bear on rhythm analysis. Resolution is judged against the daily excursion of the signal since a rhythm can only be estimated where instrument noise is small relative to the daily change; drift matters because a trend over a multi-week record biases the mean and the baseline limits more than it biases phase or normalised amplitude.

Sensor Modality	Measurement Resolution Relative to the Daily Excursion	Drift or Recalibration Over a Multi-Week Record
Physicochemical (temperature, humidity, CO_2_)	High: digital probes resolve far below the 1 to 2 °C daily excursion of the brood nest; CO_2_ sensors resolve tens of ppm against daily swings of hundreds [29,32]	Low for temperature and humidity; optical CO_2_ sensors drift over months, which shifts the mean more than the phase or the normalised amplitude
Gas sensor arrays (e-nose)	Moderate: a multivariate response with no single calibrated quantity, so rhythm must be estimated on derived scores	High: baseline drift over days to weeks and cross-sensitivity to temperature and humidity require explicit correction [40]
Hive scales	High: load cells resolve tens of grams against daily excursions of hundreds of grams to kilograms [45]	Low: load cell creep and temperature coefficient affect the level, not the daily waveform; rain and manipulations produce step artefacts that must be flagged
Infrared entrance counters	High: individual passage events	Low: beam fouling by debris and direct sunlight degrade counts and need periodic cleaning
Thermal imaging	High spatially; absolute accuracy depends on emissivity and reflected radiation	Moderate: ambient and emissivity corrections required; rarely operated continuously
Acoustic sensors	High: continuous audio; effective resolution is set by the feature extracted (band power, spectral indices)	Low for the microphone itself; environmental noise, not drift, is the limitation [66]
Vibrational sensors	High for the transducer	Installation-specific: sensitivity depends on frame material and mounting, so each installation needs its own baseline
Video (entrance)	High: behaviour-specific counts and trajectories	Low for the sensor; illumination and lens fouling vary over a deployment [87]
Video (in-hive)	High	Moderate: wax and propolis accumulate on optics, and lighting alters behaviour [86]
RFID	Event-level timing; missed reads at high traffic	Low: tag loss and reader fouling over the lifespan record
Harmonic radar	Trajectory resolution of metres	Not applicable: records span flights, not weeks
Doppler radar	Aggregate motion index without count resolution	Low: clutter from vegetation and weather varies rather than drifts [110]
Lidar	Transit counts, with wingbeat frequency resolved	Low: optical alignment is stable; sensitivity is weather-dependent
Capacitive counters	Count resolution; undercounting at high traffic [115]	Low: insensitive to light and weather; sustained baseline shift when bees cluster at the entrance
Electrostatic field (ESF)	Behaviour-level events reported [117]	Unknown: no field performance data across hive materials, colony sizes, or seasons

**Table 4:** Alert day for each detection rule in the simulated example (perturbation onset day 30; all rhythm rules computed on entrance activity; the set point rule on brood nest temperature).

Rule	Data Stream	Alert Day	Days After Onset	Days Before Set Point Alert
Relative amplitude	Entrance activity	35	5	21
Interdaily stability	Entrance activity	37	7	19
Acrophase	Entrance activity	43	13	13
Intradaily variability	Entrance activity	46	16	10
Temperature < 33 °C for 1 h	Brood nest temperature	56	26	0

## Data Availability

This review reports no primary empirical data. The script that generates the simulated dataset and the AnCiR session that reproduces the analysis for the worked example in Section 4 are available at https://doi.org/10.5281/zenodo.22884569. This study is a synthesis of monitoring technologies and an analytical framework; it does not involve primary field data collection.
